# Cancer awareness in Australian adolescents

**DOI:** 10.1186/s12889-023-16406-z

**Published:** 2023-08-01

**Authors:** F. E. J. McDonald, X. Skrabal Ross, G. Hubbard, S. Konings, A. Jeitani

**Affiliations:** 1 Research, Policy and Patient Department, GPO Box 3821, Canteen, Sydney, NSW 2001 Australia; 2 Faculty of Medicine and Health, The University of Sydney, Sydney, Australia; 3 Department of Nursing and Midwifery, University of the Highlands and Islands, Scotland, Inverness, UK; 4 Psycho-Oncology Department, Clinique Saint-Jean, Bruxelles, Belgium

**Keywords:** Cancer, Public cancer awareness, Early diagnosis, Help seeking behaviour, Adolescents

## Abstract

**Background:**

Over one-third of cancer cases are attributable to modifiable risk factors. Because health-related behaviors are often established at adolescence, it is important that adolescents understand the risks and lifestyle decisions that may reduce their chances of developing cancer. This study aims to identify the levels of cancer awareness of adolescents in Australia.

**Methods:**

Paper questionnaires were used to collect information about baseline levels of cancer awareness. These questionnaires included socio-demographic questions and the Cancer Awareness Measure (CAM) with slight modifications to ensure their suitability for the Australian adolescent population. Students aged 11 to 19 years were recruited from 13 Australian high schools between 2016 and 2019.

**Results:**

A total of 766 adolescents (58% female, mean age = 14.5 years) completed the questionnaires. Adolescents’ cancer awareness was low. Adolescents who knew someone with cancer recognized significantly more cancer risk factors and cancer warning signs than those who did not know someone with cancer (t (756) = 2.35, p = .019; t (747) = 5.57, p = .001). Those from high Index of Community Socio-Educational Advantage (ICSEA) schools significantly recognized more cancer risk factors than those from low ICSEA schools (t (764) = 2.42, p = .016). Females recognized significantly more warning signs than males (t (583) = 3.11, p = .002) and students from senior high school grades recognized more warning signs than those from junior grades (t (754) = 2.24, p = .02). Most adolescents (78%) were aware of skin cancer as one of the most common cancers in Australia, however half or less were aware of other common cancers. Although most adolescents would seek medical help in the presence of possible cancer symptoms as soon as possible, approximately 20% of them would not see a doctor promptly. Emotional barriers were the most common reasons to delay seeing a doctor (56%), for example “being worried about hearing bad news” (27%).

**Conclusions:**

Australian adolescents show poor awareness of cancer risk factors and cancer warning signs. A number of demographic and experience factors were found to be related to lower cancer awareness. Education is essential to raise cancer awareness, promote healthy lifestyles from adolescence and avoid a preventable cancer diagnosis.

## Background

In 2022, around 162,000 new cancer cases were expected to be reported in Australia, with this number rising yearly [1]. Over one-third of these cases are attributable to modifiable risk factors such as smoking, obesity, drinking alcohol, poor physical activity, unhealthy diet, and exposure to the sun that can be prevented through lifestyle changes [2]. Unhealthy lifestyle behaviors can emerge during adolescence [3, 4] and are likely to continue into adulthood [5]. In Australia, 67% of adults and 25% of children and adolescents are obese or overweight [6] and one in four Australians consume alcohol in harmful quantities [7].

Adolescence is a key window of development wherein individuals undergo physical, psychological, social, and educational changes. Throughout this period, responsibility for health issues shifts from the parents to the adolescents and health-related behaviors that continue into adulthood are often established during this phase [8]. There is an association between awareness of cancer risk factors amongst adolescents and the development of protective health behaviors that can establish the foundations for healthy adulthood [9]. It is important for adolescents to understand risks associated with their behaviors and the lifestyle decisions they can make to reduce their chances of developing cancer.

While early detection of cancer can lead to timely treatment and cure [10], lack of awareness of common cancer warning signs or symptoms is associated with delays in seeking medical help in adults [11] and adolescents [12]. Awareness of these warning signs during adolescence is critical to improve cancer prevention and early diagnosis. It is also important to understand when and why individuals would seek help if they detected a potential warning sign of cancer.

Efforts to benchmark levels of cancer awareness in adolescents are limited, and to our knowledge, there is a lack of evidence on Australian adolescents. Evidence shows that the levels of cancer awareness in adolescents and young adults in the UK and Turkey for instance, are quite low, especially in those who have not received cancer education [9, 13] or have not experienced cancer in their family [14]. Adolescents in Canada and the UK are aware of the cancer-related risks associated with smoking, but often fail to identify the risks associated with poor diet and lack of physical activity [9, 15]. Further, cross-cultural differences have been observed in adolescents’ recognition of cancer warning signs. While there is higher recognition of “a sore that does not heal” as a warning sign amongst Omani adolescents compared to those in the UK (39% vs. 24%-26%), those in the UK show higher recognition of “changes in bowel and bladder habits” than Omani adolescents (53-54% vs. 28%) [12, 16, 17]. There are also cross-cultural similarities amongst Omani, and UK adolescents in the recognition of the presence of an unexplained lump or swelling as a warning sign of cancer and the most common barriers to seeking help being emotionally related and, in particular, worrying about what the doctor might find [12, 16, 17].

This study is part of a larger wait-list control study evaluating the effectiveness and acceptability of a cancer education program for Australian secondary school students titled *When Cancer Comes Along*. The program, developed and delivered by Canteen^1^, was designed to increase adolescents’ awareness of cancer including what cancer is, how to lower their risk of cancer later in life, how cancer impacts peoples’ lives and how they can help a friend impacted by cancer. Adolescents who have completed similar programs in the UK show greater awareness of cancer risk factors and warning signs [13]. The current paper focuses on adolescents’ pre-existing cancer awareness before undertaking the program.

This study aimed to identify the levels of cancer awareness of adolescents in Australia including their knowledge of common cancers, cancer risk factors, cancer warning signs, and cancer-related help-seeking behaviors. Findings from this study are useful for comparisons of cancer awareness internationally and against adults, and for identifying the impact of and gaps in existing Australian health promotion campaigns and education programs directed towards adolescents.

## Methods

### Sample

Data were collected from students aged 11 to 19 years, recruited from 13 Australian high schools between 2016 and 2019. Schools were recruited into the study following promotion of the program on social media, conferences, and via word of mouth from Canteen staff. A convenience sampling approach was employed by recruiting amongst schools whose staff had existing relationships with Canteen staff. The characteristics of the schools are described in the Results section.

### Data Collection

Teachers administered paper questionnaires which were completed individually by students and measured baseline levels of cancer awareness (approximate completion time was 15–20 min). Questionnaires incorporated sociodemographic questions such as: age, gender, country of birth, parents’ countries of birth, Aboriginal and Torres Strait Islander status, and whether students knew someone who had been diagnosed with cancer. Each student was assigned with an Index of Community Socio-Educational Advantage (ICSEA) [18] value which reflects their school’s educational advantage, measured using the occupation and education of students’ parents. Students from the same school were assigned the same ICSEA value.

Questionnaires also included the Cancer Awareness Measure (CAM) [19] which is a widely used validated measure of cancer awareness and help-seeking behaviors. With only minimal modifications needed to ensure its face validity, it was previously adapted for use with an adolescent population [12]. The current study uses the CAM questions with slight modifications to ensure their suitability for the Australian adolescent population. Table 1 shows an outline of the questions and modifications. To ascertain level of awareness around cancer risk factors, a ‘recall then recognise’ question format was used. First, students recalled any cancer risk factors (open-ended question). They were then given a list of potential risk factors and indicated how much they agreed (or disagreed) that each item on the list was indeed a risk factor for cancer. A similar ‘recall then recognise’ question format was used to ascertain level of knowledge for cancer warning signs. The questionnaire used in this study also included questions about delay and barriers to professional help-seeking in the presence of a suspected cancer symptom, the most common cancers in Australia, and the relationship between cancer and age.

**Table 1:** Outline of the Cancer Awareness Measure

Variable	Prompt	Number of items	Response scale	Example item	Modification from original CAM
Risk factors					
Recall (open-ended)	“What things do you think affect a person’s chance of developing cancer?”	-1	-	-	-
Recognition (closed)	“How much do you agree that each of these can increase a person’s chance of developing cancers?”	10 items	5-point Likert scale (strongly disagree to strongly agree)	“Smoking any cigarettes at all”	-
Age Related to Cancer	“At what age is someone most likely to develop cancer?”	1 item	20s, 30s, 40s, 50s, 60s, 70s, 80s, Cancer is unrelated to age	-	-
Common Cancers	“What do you think are the most common cancers in Australia?”	-1	-	-	Original CAM asks about most common cancers in females and males separately and does not specify the country.
Warning Signs					
Recall (open-ended)	“There are many warning signs and symptoms of cancer. Please name as many as you can think of:”	-	-	-	-
Recognition (closed)	“Do you think the following symptoms could be a sign of cancer?”	9 items	Yes/ No/ I don’t know	“An unexplained lump or swelling”	-
Help-Seeking					
Why Delay	Open-ended: “Sometimes people put off going to see the doctor, even when they have a symptom that they think might be serious. What might put you off going to see the doctor?”	-	-	-	Original CAM lists 10 potential reasons for delay with yes/no/don’t know ratings followed by the option to add other reasons rather than an open-ended question only.
When to seek Help	“If you had a symptom that you thought might be a sign of cancer how soon would you contact your doctor to make an appointment to discuss it?”	-	-	-	-

Recall (open-ended) and recognition (close-ended) questions were used in this study to explore cancer awareness in adolescents using two different memory processes. The use of both types of questions allows to describe cancer awareness levels based on the presence (recognition) or absence (recall) of cues or prompts [20], and to gather additional qualitative data which cannot be collected through close-ended questions. There is evidence that recognition of correct answers tends to be higher than recall when testing students [21]. Differences in findings between the two types of questions may help to inform education strategies to facilitate cancer awareness in adolescents.

### Analysis

Data were analysed using IBM SPSS Statistics 25. Descriptive statistics were used for demographics (e.g., gender, age, having at least one parent born overseas, Aboriginal/Torres Strait Islander status, knowing someone with cancer, ICSEA index), and CAM items. Bivariate statistical analyses for the demographic variables (gender = male/female, know someone with cancer = yes/no, school’s ICSEA percentile = high > 50/low < 50, and grade = junior [7–9] /senior [10–12]) and two dependent variables (awareness of cancer risk factors and awareness of cancer warning signs) were conducted using t-tests, followed by multiple regression analyses which included all the demographic variables described above. Qualitative data on reasons why adolescents might put off going to see a doctor was coded according to the 10 reasons provided in the CAM; categorised as Emotional, Practical or Services barriers.

Open-ended responses left blank were included in ‘I don’t know’ categories. All unanswered multiple-choice (recognition) responses were excluded from analysis.

## Results

### Sample

A total of 766 adolescents (female: n = 442, 58%) aged between 11 and 19 years old (mean age = 14.5, SD = 0.93) from 13 high schools across Australia completed the baseline survey. The composition of the participating high schools was as follows: one from Western Australia, one from Queensland, six from New South Wales, three from Victoria and two from Tasmania; 318 (41%) adolescents were from six government schools, 349 (46%) adolescents were from five independent schools, and 99 (13%) adolescents were from two catholic schools.

Adolescents’ demographic characteristics are presented in Table 2. Most of the adolescents knew someone with cancer (n = 550/758, 73%) and 127 (23%) noted more than one person. From the adolescents who knew someone with cancer, common responses included a grandparent (n = 211, 38%), other family member (e.g., aunt, uncle, cousin; n = 167, 30%), family friend (n = 155, 28%), parents (n = 65, 12%), or friend/peer (n = 51, 10%). Only 2% stated sibling, pet or themselves.

**Table 2:** Sample Demographic Characteristics

Demographics	n	%
*Gender*		
Male	310	40
Female	442	58
Other	8	1
Missing	6	1
*Age*		
11	1	0.1
12	8	1
13	85	11
14	240	31
15	333	43
16	81	11
17	6	1
18	1	0.1
19	2	0.2
Missing	9	1
*Grade level*		
Junior (7–9)	509	66
Senior (10–12)	257	34
*Country of birth*		
Australia	667	87
Other	94	12
*Parents Country of Birth*		
Both born in Australia	445	58
At least one born overseas	284	37
Missing	37	5
*Of Aboriginal or Torres Strait Islander Descent*		
Yes	52	7
No	705	92
Missing	9	1
*Knew Someone with Cancer*		
Yes	550	72
No	208	27
Missing	8	1
*Index of Community Socio-Educational Advantage (ICSEA)*		
High ICSEA	445	58
Low ICSEA	321	42

## Cancer Risk factors

**Fig. 1:**
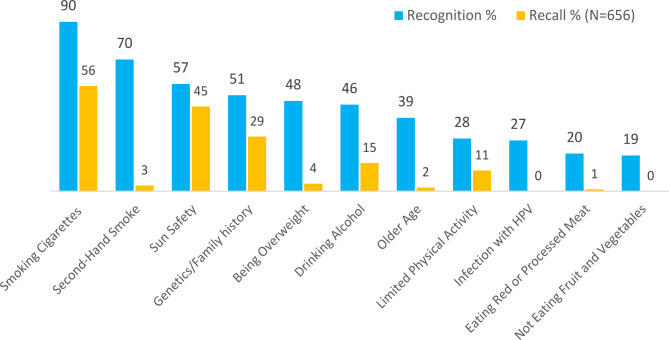
Recall and Recognition of Cancer Risk Factors NOTE - Recognition options were given as follows: (1) Smoking any cigarettes at all; (2) Exposure to another person’s cigarette smoke; (3) Drinking more than 1 unit of alcohol a day; (4) Eating less than 2 portions of fruit and 5 vegetables a day; (5) Eating red or processed meat once a day or more; (6) Being overweight (BMI over 25); (7) Getting sunburnt more than once as a child; (8) Being over 70 years old; (9) Having a close relative with cancer; (10) Infection with HPV (Human Papillomavirus); (11) Doing less than 30 min of moderate physical activity 5 times a week

Figure 1 shows rates of recognition and recall of 11 common cancer risk factors. In all cases, the recall of cancer risk factors (open-ended question) was noticeably lower compared to the recognition of these factors, when presented with the response options. The most frequently recognized and recalled risk factors were smoking cigarettes and those related to sun safety. Notably, while 173 (26%) students recalled diet as a risk factor for cancer, very few (less than 2%) recalled specific foods (e.g., high consumption of processed red or meats, or low consumption of fruit and vegetables). In contrast, students were specific about sun safety risks with sun exposure, sunburn or tanning being recalled 194 times (30%), and not applying sunscreen, not wearing protective clothing or not being sun smart (including applying sunscreen, wearing protective clothing and seeking shade) being recalled 126 times (19%). One in four students stated lifestyle as a cancer risk factor (n = 149, 23%). Other responses to the open-ended question included drugs (n = 46, 7%), the environment (n = 40, 6%), radiation (n = 32, 5%), lack of medical check-ups (n = 24, 4%), and chemical exposure (n = 21, 3%) as risk factors. All other risk factors provided by students were recalled in fewer than 15 responses (e.g., stress, luck, cancer knowledge, skin colour). One hundred and fifty-six students (20%) in response to the open-ended question did not know a risk factor of cancer (n = 113; 15% left the question blank and n = 43; 5% stated that they ‘do not know’ risk factors of cancer).

The mean number of risk factors of cancer recognised (closed-ended question) was 4.67 (SD = 2.36). Bivariate statistical analyses revealed that adolescents who knew someone with cancer recognized significantly more cancer risk factors (Mean = 4.82, SD = 2.35) than those who did not know someone with cancer (Mean = 4.37, SD = 2.36; t (756) = 2.35, p = .019). Students from high ICSEA schools recognized significantly more cancer risk factors (Mean = 4.84, SD = 2.37) than those from low ICSEA schools (Mean = 4.43, SD = 2.33; t (764) = 2.42, p = .016). No significant difference in the number of risk factors recognised emerged between females and males or between grade levels (junior and senior).

Consistent findings were obtained when all four demographic variables (knowing someone with cancer, ICSEA group, gender, and grade level) were included as independent variables in a multivariate regression analysis to predict recognition of cancer risk factors. The overall regression was statistically significant (F (4,748) = 3.50, p = .008) but only explained 1% of the variance in the dependent variable (adjusted R square = 0.013). ICSEA group (β = 0.39, p = .030) and knowing someone with cancer (β = 0.42, p = .030) significantly predicted recognition of cancer risk factors when controlling for the other demographic variables.

### Cancer warning signs

**Fig. 2:**
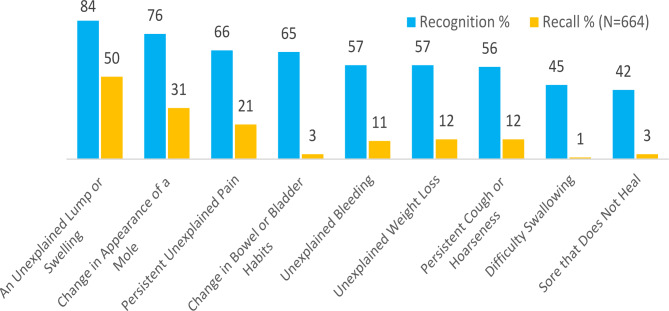
Recognition and Recall of the Most Common Cancer Warning Signs

Figure 2 shows rates of recognition and recall of the nine most common cancer warning signs. As was the case with cancer risk factors, adolescents’ recall of cancer warning signs (open-ended question) was low, compared to their recognition of these signs (when the written options were provided). The mean number of cancer warning signs recognised was 5.56 (SD = 2.40). The most recognized and recalled warning signs of cancer were “an unexplained lump or swelling” (84% and 50%, respectively), followed by “change in appearance of a mole” (76% and 31%, respectively), and “persistent unexplained pain” (66% and 21%, respectively).

More than one-third of the adolescents who answered the question did not recall any of the nine most common warning signs; their answers did not align with any of the most common warning signs (n = 184, 28%) or stated that they did not know (7%). Other responses to the recall question about warning signs included hair loss (n = 97, 13%), coughing blood (n = 89, 12%), fatigue (n = 86, 11%), generally unwell (n = 85, 11%), spots on skin (n = 71, 9%), shortness of breath (n = 58, 8%), swelling (n = 56, 7%), sickness (n = 53, 7%) and skin discolouration (n = 47, 6%).

Bivariate analyses revealed that females recognized significantly more warning signs than males (5.81, SD = 2.20 vs. 5.24, SD = 2.59; t (583) = 3.11, p = .002). Students who knew someone with cancer recognized significantly more cancer warning signs than those who did not know someone with cancer (5.85, SD = 2.26 vs. 4.76, SD = 2.61; t (747) = 5.57, p = .001). There was a significant relationship between grade level (junior and senior) and recognition of warning signs, with higher recognition in senior grades (5.42, SD = 2.42 vs. 5.83, SD = 2.84; t (754) = 2.24, p = .02). No significant difference emerged between adolescents from high and low ICSEA schools.

Consistent findings were obtained when all four demographic variables (knowing someone with cancer, ICSEA group, gender, and grade level) were included as independent variables in a multivariate regression analysis to predict recognition of cancer warning signs. The overall regression was statistically significant (F (4,744) = 12.20, p = < 0.001) but only explained 6% of the variance in the dependent variable (adjusted R square = 0.057). Gender (β = 0.57, p = < 0.001), knowing someone with cancer (β = 0.98, p = < 0.001), and grade level (β = 0.36, p = .047) significantly predicted recognition of cancer warning signs when controlling for the other demographic variables.

### Relationship between Cancer and Age

Of the 696 adolescents who answered the question “at what age people are most likely to develop cancer?”, 392 (56%) stated that cancer was unrelated to age, 157 (23%) stated people in their 20s, 30s, 40 or 50 s, and 147 (21%) stated people in their 60s, 70 or 80s .

### Common cancers in Australia

The five most common responses from students who answered this recall (open-ended) question (N = 684) were in line with the five most common cancers in Australia: skin^2^ (n = 533, 78%), breast (n = 370, 54%), lung (n = 355, 53%), bowel, (n = 120, 18%), and prostate (n = 61, 9%). Although most adolescents were aware of skin cancer, half or more of the adolescents were not aware of the rest of the five common cancers in Australia, with awareness of bowel and prostate cancer being extremely low in comparison. Additional recall (open-ended) responses included brain (n = 47, 7%), leukemia (n = 34, 5%), liver (n = 33, 5%), cervical (n = 24, 4%), throat (n = 23, 3%), and blood (n = 16, 2%). Out of 766 participants, 106 (14%) did not provide a response about the most common cancers (83 left the question blank and 23 stated they did not know).

### Anticipated Delay to seek help

When participants were asked when they would see the doctor in the presence of a possible sign of cancer, most of the adolescents who answered the question stated ‘as soon as possible’ (n = 518/714, 73%) and an additional 12% (n = 85) of the adolescents said that they would see a doctor relatively soon (within a few days to two weeks). The other 15% of the adolescents provided diverse responses, for example, “within one month”, “never” or “if it gets worse”.

### Barriers to help-seeking

Barriers categorized in the CAM measure and additional barriers that did not fit within CAM’s categories were identified. Amongst adolescents who completed this question (N = 698), Emotional barriers were the most common reasons to delay seeing a doctor (n = 390, 56%). In this category the main reasons to delay seeing a doctor were that they might be worried about hearing bad news (n = 190, 27%), too scared (n = 101, 15%), or too embarrassed (n = 70, 10%). The second most common barriers (not present in the CAM categories) were Cognitive barriers (n = 192, 28%). Not thinking the symptom is serious and it will go away (n = 121, 17%) and denial (n = 50, 7%) were the most cited reasons in this category. The third most common type of barriers were Practical barriers (n = 186, 27%). The most common reasons in this category were that they might be too busy (n = 77, 11%) or money (n = 50, 7%). Few students (n = 48, 7%) stated Service barriers as potential reasons for not seeing a doctor. Thirty-three students (5%) stated it might be difficult to talk to a doctor or that they do not like to talk to doctors.

## Discussion

This study contributes to the worldwide emerging literature on adolescent cancer awareness. Findings reveal low levels of cancer awareness in adolescent Australians and highlight the need for future awareness initiatives with this population. The inclusion of recall data in the current study offers some further insight into the awareness of cancer risk factors and warning signs, showing that when adolescents were not presented with response options their rates of recall were noticeably lower than their rates of recognition. This is consistent with previous evidence on lower recall rates compared to recognition rates when students’ knowledge is tested [21].

The Australian National Action Plan for the Health of Children and Young People: 2020–2030 [22] highlights the need to implement health strategies that address risk factors for chronic diseases, prevent risky behaviors in adolescents, and improve access to health services for children and young people. Findings from this study may help to inform cancer-related initiatives for adolescents in line with the National Action Plan.

### Cancer Risk factors and Cancer warning signs

Findings from this study show a low level of awareness about cancer risk factors and warning signs amongst adolescent Australians. Consistent with other countries around the world, where the use of tobacco is the most identified cancer risk factor amongst adolescents [9, 16, 23], smoking cigarettes and second-hand smoke were the most recognized amongst adolescent Australians (90% and 70%, respectively). National anti-tobacco campaigns in Australia started in 1972 and have proven to be effective in reducing the prevalence of smoking amongst adolescents [24].

The most common cancer amongst young Australians is melanoma (a form of skin cancer) and Australia has the highest incidence of melanoma in the world [25]. Despite this, approximately half of the sample of adolescents in this study did not recognize ‘getting sunburnt’ as a common cancer risk factor, with even lower recall of sun-related risks when the answer option was not provided in the survey. This is an unexpected finding considering the extensive reach of sun protection awareness campaigns from the Australian SunSmart Program which started in 1988 and continues to oversee the implementation of sun protection policies at childcare centres and primary schools [26]. It is possible that many of the adolescents in this study were aware of the relation between not using sun protection (e.g. hat, sunscreen) and skin cancer but were not aware of the relation between getting sunburnt and cancer. Blistering sunburns increase the risk of developing both melanoma and non-melanoma skin cancer [27, 28]. While sun safety is not practiced by many children, the ability to achieve long-term increase in sun protective behavior from educational initiatives exists [29].

In this study, there was a poor recognition and recall of diet-related cancer risk factors, including eating read or processed meats and not eating enough fruits or vegetables. Moreover, less than 50% the Australian adolescents recognized ‘being overweight’ as a cancer risk factor. Previous studies show that the recognition of diet-related cancer risk factors amongst Australian adults is also low and up to 60% of the adults are not aware that not eating enough fruits and vegetables and eating red or processed red meats are cancer risk factors [30–32].

Finally, less than half of the adolescents in this study recognized drinking alcohol and limited physical activity as common cancer risk factors. Along with poor nutrition (including diets low in fruits and vegetables) these are also major risk factors for chronic diseases such as diabetes and heart disease [33]. This highlights the need for health promotion activities and education that increase adolescents’ health literacy (e.g., risk of drinking alcohol campaigns and specific dietary behaviors related to cancer prevention from young ages).

Similar to Australian adolescents, low levels of cancer awareness have been observed amongst UK adolescents [9, 12]. The presence of similarities in Australian and UK adolescents’ awareness of a number of cancer risk factors (e.g., high awareness of smoking cigarettes and poor awareness of eating red processed meats and not eating enough vegetables and fruits) and warning signs (e.g., high awareness of an unexplained lump or swelling and poor awareness of a sore that doesn’t heal) may be the result of exposure to similar educational or awareness campaigns. However, there are differences between Australian and UK adolescents in levels of awareness of a number of cancer risk factors (e.g. being overweight) and cancer warning signs (e.g. an unexplained lump or swelling, persistent unexplained pain) which may indicate the need for different approaches to policy and interventions to address awareness deficits in adolescents from different geographical and cultural contexts.

### Knowledge of Cancer Prevalence in Australia

Most adolescent Australians are aware of skin cancer as one of the most common cancers in Australia and approximately half of them are aware of breast and lung cancers. However, there is poor awareness of bowel and prostate cancers. The strong focus of Australian national campaigns on sun protection behaviors, breast cancer awareness and tobacco control may explain these findings and the poor awareness of adolescents about bowel and prostate cancers. Breast cancer is the cancer with the highest incidence in Australia, with prostate and bowel cancer in the second place, followed by skin cancer (melanoma) [34]. Melanoma is the cancer with highest incidence in Australian young people [25].

Similar to UK adolescents, approximately half of Australian adolescents consider that cancer is unrelated to age, with low awareness that older people (60–80 y/o) are at a higher risk of developing cancer compared to other age groups.

### Socio-Demographic factors and Cancer Awareness

Most of the adolescents in this study knew someone with cancer. Knowing someone with cancer was associated with higher levels of recognition of warning signs of cancer, mirroring the UK study samples of adolescents [12, 17]. In this study, knowing someone with cancer was also associated with higher recognition of cancer risk factors. This highlights the need to consider tailored cancer education targeting adolescents who have not been exposed to a close cancer experience.

In this research, gender was found to have a significant relation with the recognition of cancer warning signs (higher recognition in females). Previous studies show a similar gender disparity in symptom knowledge between Australian adults regarding lung cancer [35] as well as between Australian adolescents and young adults regarding symptoms of mental health issues [36].

Students from senior grades [10–12] significantly recognized more cancer warning signs than those from junior grades but no association was found between grade level and recognition of cancer risk factors. While the first could be explained by senior students’ higher exposure to academic content related to health and possibly cancer, it is not clear why senior students were not better than junior students at recognizing cancer risk factors.

Compared to low ICSEA schools, high ICSEA schools recognised significantly more cancer risk factors. Although high ICSEA schools also recognized more cancer warning signs, this difference was not significant. In Australia there are no significant differences in the incidence of cancer between people from low and highs socioeconomic status. Exemptions to this are lung cancer, with higher incidence in people from a lower socioeconomic status, and breast and prostate cancer with higher incidence in people from less disadvantaged socioeconomic status. However, those from disadvantaged socioeconomic status are likely to survive less years through cancer than those from less disadvantaged socioeconomic status [37].

### Barriers to help-seeking

While there is extensive literature on reasons why adults delay seeking medical help, there is limited understanding on reasons for adolescents. Aligning with the UK studies with adolescents [12, 17], findings from this research indicate that Emotional barriers, especially being worried about hearing bad news, or being too scared or embarrassed, are the main reasons why adolescents delay seeking medical attention in the presence of a possible cancer symptom. This is an interesting finding considering that this study used an open-ended question to replace the multiple option item (with 10 options) used in the CAM measure to assess help-seeking barriers.

Importantly, the second most common reason for delay in help-seeking, which does not fit into any of the three pre-existing barrier groups (in the CAM measure), was assuming the symptom was not serious (Cognitive barrier). This suggests the need for further education for adolescents on the seriousness of cancer warning signs to prevent delays in diagnosis [38]. Very few Australian adolescents in this study stated service barriers compared to a study with UK adolescents where more than one quarter of the participants identified service barriers to cancer-related help-seeking (e.g., difficulties making an appointment or talking to the doctor) [12]. This suggests that these barriers might not be the primary concerns of Australian adolescents.

The open-ended nature of the question about help-seeking barriers in this study reveals other emotional barriers (e.g., disliking medical procedures and doctors), cognitive barriers (e.g., denial) and practical barriers (e.g., money) that were not identified by the UK study [12].

### Future Research Priorities

The current study points to avenues for future research. Future studies should purposefully recruit adolescents in different age groups to explore age-related differences in cancer awareness and help-seeking behaviors. Similarly, studies conducted in other countries would enable comparisons between adolescent populations internationally and may reveal differences in education or the effectiveness of national health campaigns. As the present study suggests, these studies may benefit from including open-ended (recall) questions on barriers to help-seeking to explore the presence of specific system or country related barriers.

The present study provides evidence on the levels of cancer awareness and help-seeking behaviors of Australian adolescents. These findings are valuable to inform the development of future cancer awareness interventions for adolescents. There is also a need for the effectiveness of these interventions to be evaluated. The current study is part of a larger study by Canteen looking at the effectiveness of a cancer awareness program for secondary students called *When Cancer Comes Along*. Cancer awareness programs for adolescents have demonstrated their effectiveness around the world [9, 23, 39].

Future studies should purposefully include vulnerable groups of adolescents such as Aboriginal and Torres Strait Islander Peoples. Aboriginal and Torres Strait Islander Peoples are 1.4 times more likely to die from cancers associated with preventable risk factors than non-indigenous Australians [40]. Higher levels of smoking and risky drinking may contribute to this disparity [41] which highlights the need for education about cancer risk factors and prevention in this population.

### Strengths and Limitations

One strength of the present study was the use of the validated CAM, as has been used in similar previous studies with adolescents. This allowed for some general comparisons to be made between Australian and UK samples. More importantly, it has established a baseline which can be used to compare with adult populations and to assess the effectiveness of intervention strategies.

Other key strengths of the study are the variety of schools including government, catholic, and independent from different Australian states, and the large sample size. That said, albeit large, the sample disproportionately represents the population of year 9 and year 10 high school students in Australia and so care should be taken when generalizing findings from this study to other age groups. Specifically, three-quarters of the students in this research were aged 14 or 15 years, and so other ages could be under-represented. Future studies with a sample of evenly distributed ages could explore differences in the levels of cancer awareness between younger and older adolescents.

In this study, the open questions exploring adolescents’ help-seeking behaviors asked about how soon they would contact the doctor and what could delay them from seeing the doctor if they experienced a suspected cancer-related symptom. However, it is more likely that young adolescents talk to their parents first about the symptom and that the parents are the ones responsible for making the decisions about contacting the doctor. We recommend future studies to explore help-seeking communication behaviors between young adolescents and their parents.

The two States and Territories with higher incidence of skin cancer in Australia were underrepresented in this study (Queensland = 22, 3% participants and the Northern Territory = 0 participants). It is possible that due to their higher incidence of skin cancer, adolescents’ awareness of skin cancer risk factors and warning signs is higher in those States, compared to the sample in this study which mostly included adolescents from States where skin cancer rates are below the national average. Future studies in Australia should purposively include adolescents from Queensland and the Northern Territory to explore differences in skin cancer awareness with adolescents from other States.

## Conclusion

Australian adolescents show poor awareness of cancer risk factors and cancer warning signs. Those who have not been exposed to a close cancer experience, males, those attending schools with low ICSEA scores and those from junior school grades show lower levels of awareness. Many of the common cancer risk factors are also risk factors for other chronic diseases. Education on these risks and protective behaviors is essential to promote healthy lifestyles from adolescence and avoid a preventable cancer or chronic disease diagnosis. Early detection of cancer can lead to timely treatment and cure. There is need for interventions to increase adolescents’ awareness of common cancer warning signs that address the most cited reasons to delay seeing the doctor in the presence of a cancer symptom: being worried about hearing bad news and thinking the symptom is not serious. Findings from this study may help to inform initiatives for cancer prevention and early detection in line with the Australian National Action Plan for the Health of Children and Young People: 2020–2030.

## Data Availability

The datasets generated and analyzed during the current study are not publicly available due to containing information that could compromise the privacy of research participants but are available from the corresponding author on reasonable request.

## Footnotes

1 Canteen Australia is a national organization that provides psychosocial support to adolescents and young adults (12–25 y/o) impacted by cancer (themselves or in their families).

2 Responses included “skin cancer”, “sun cancer” and “melanoma”.
